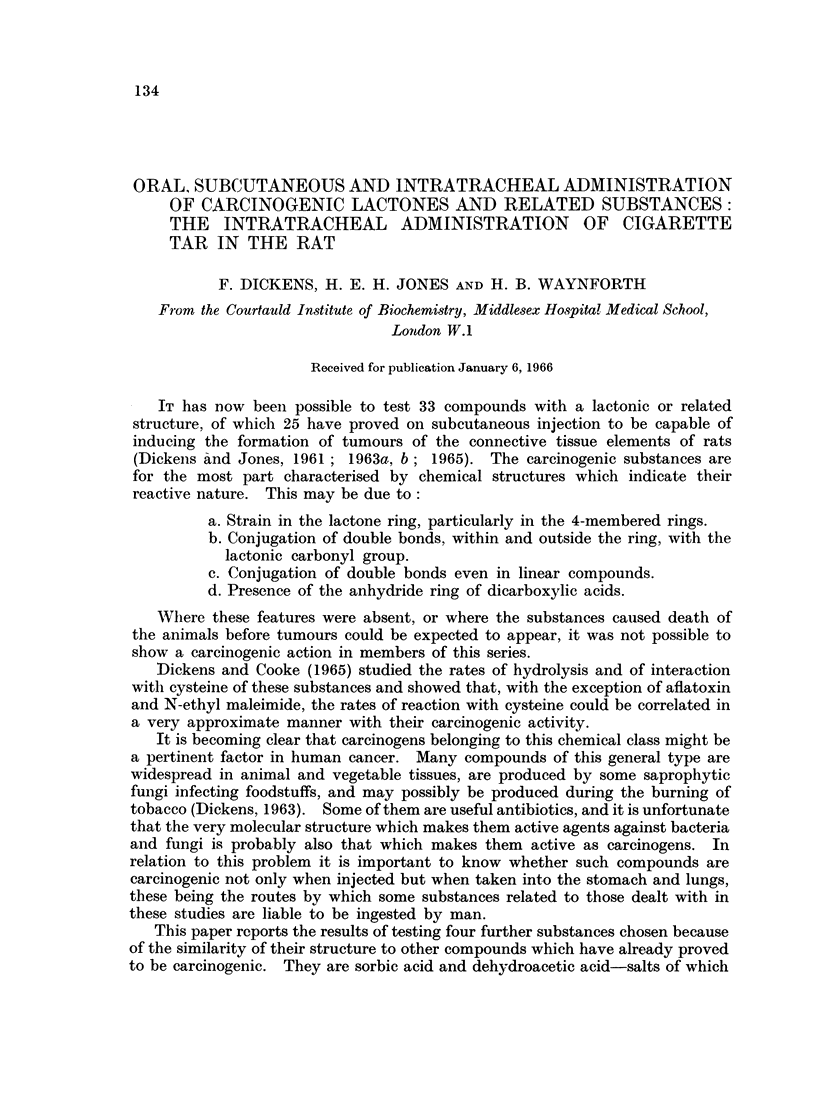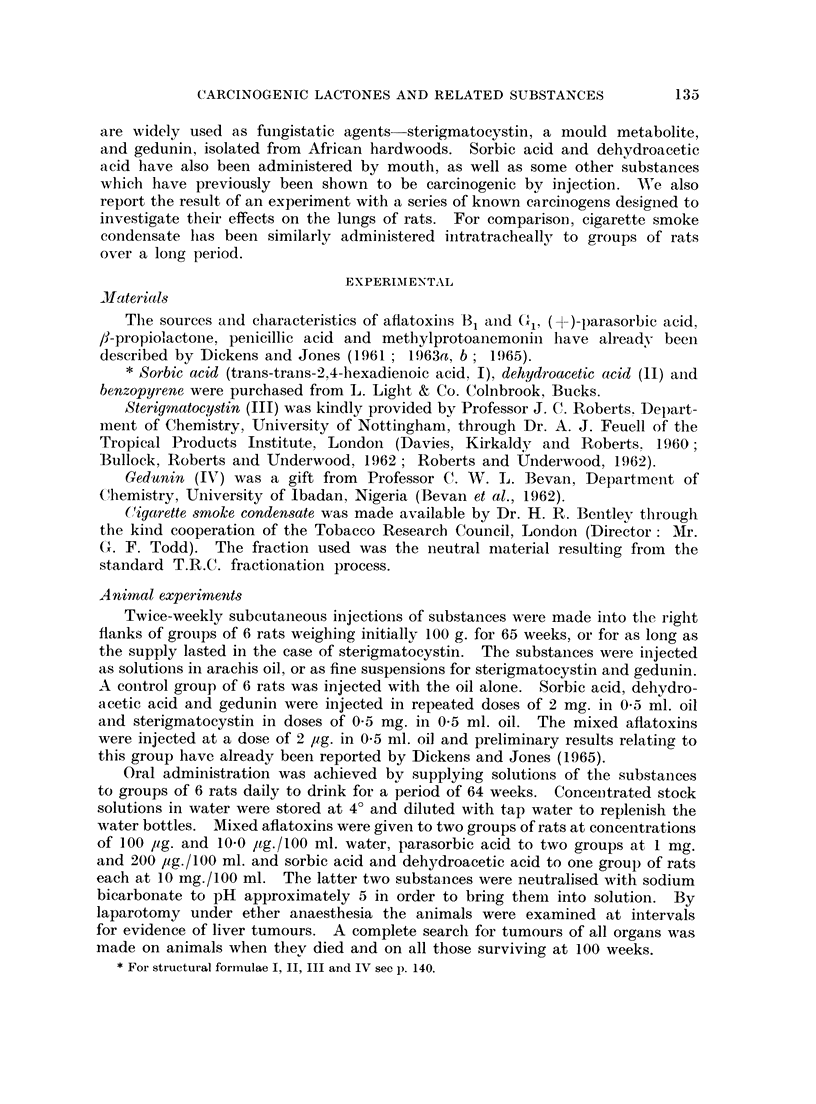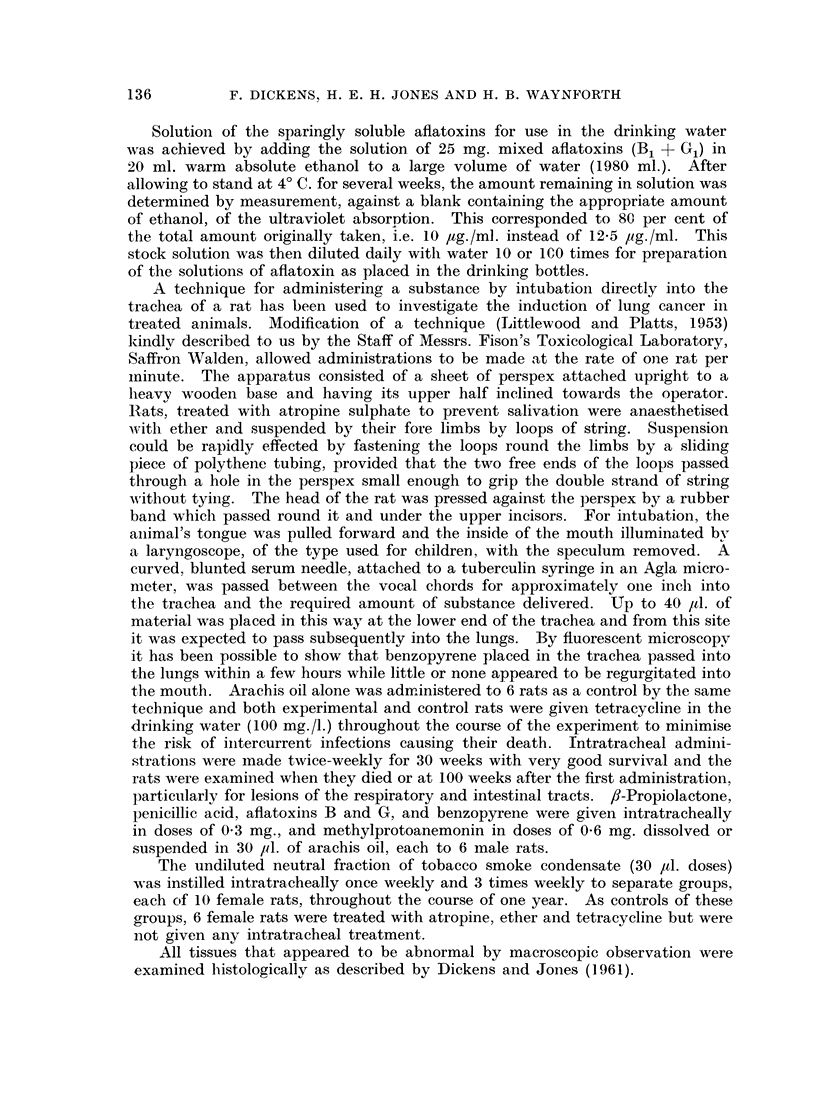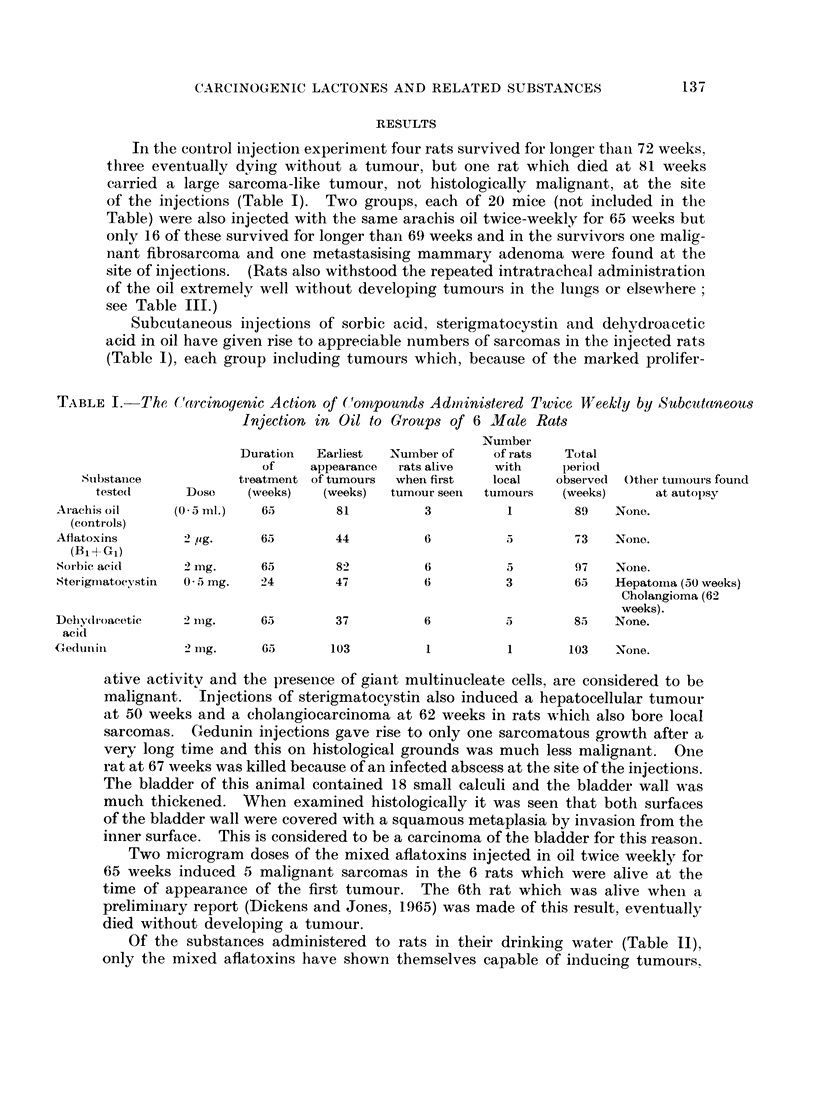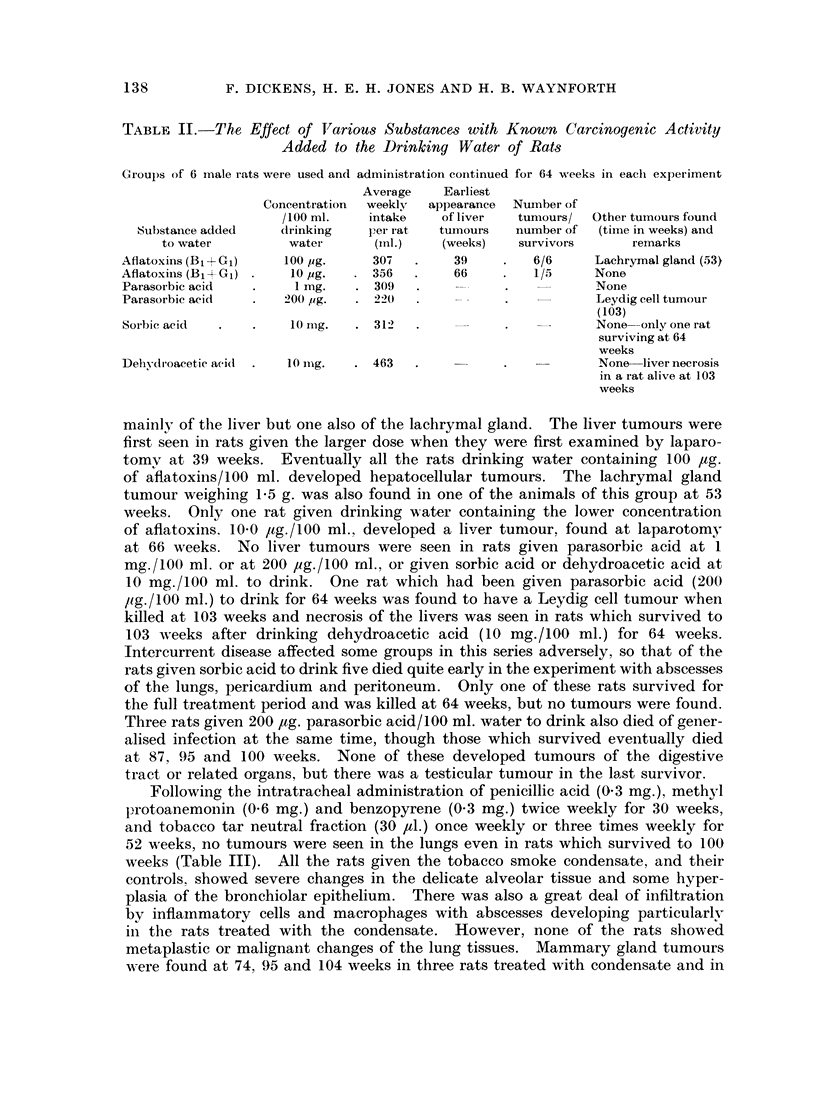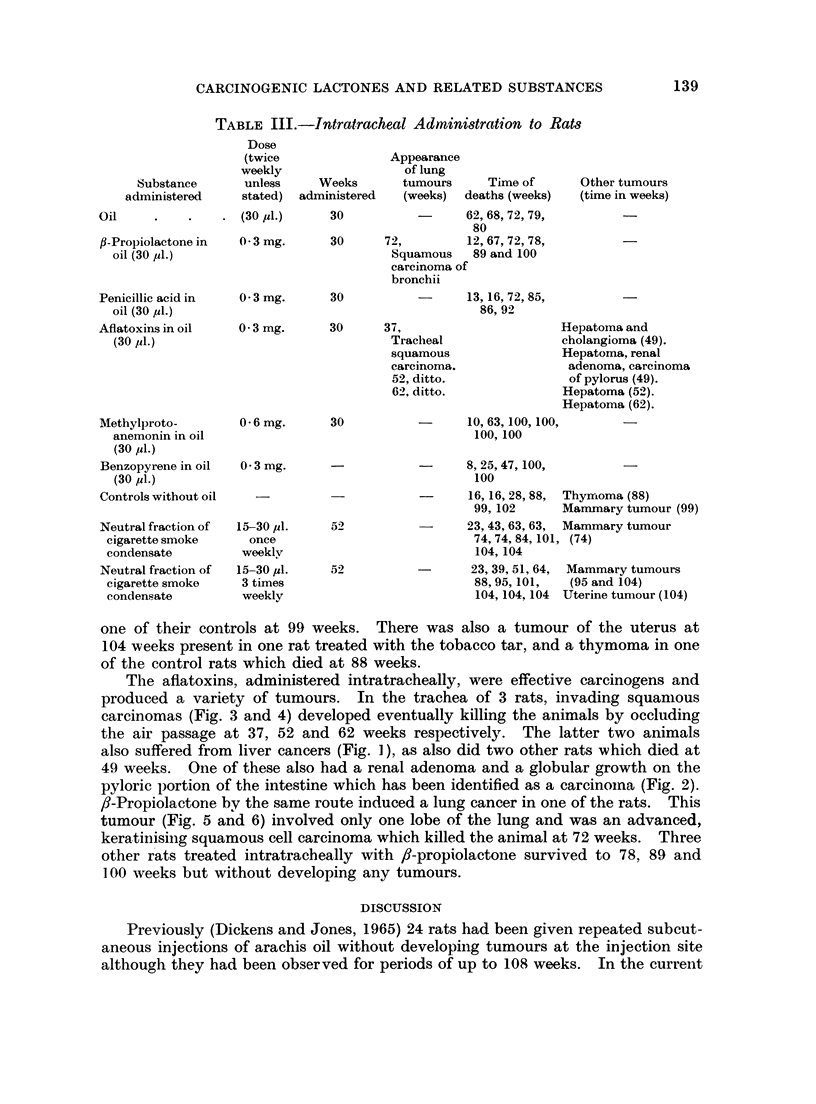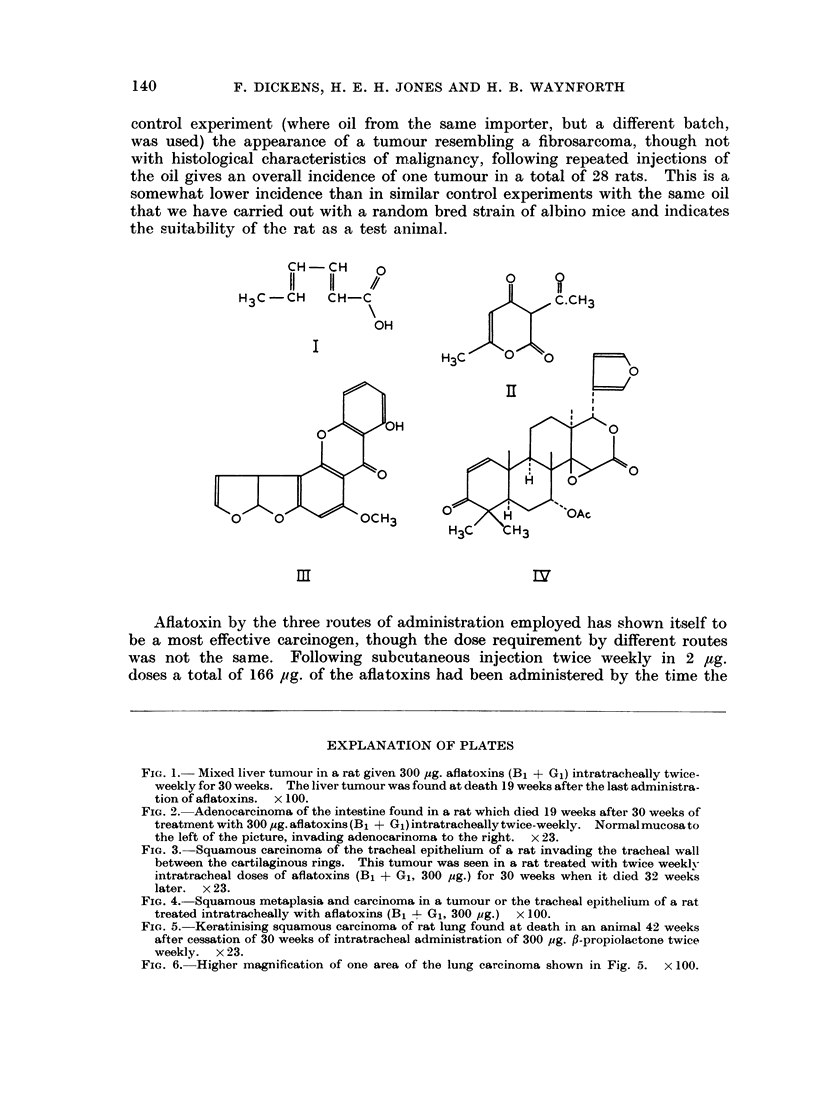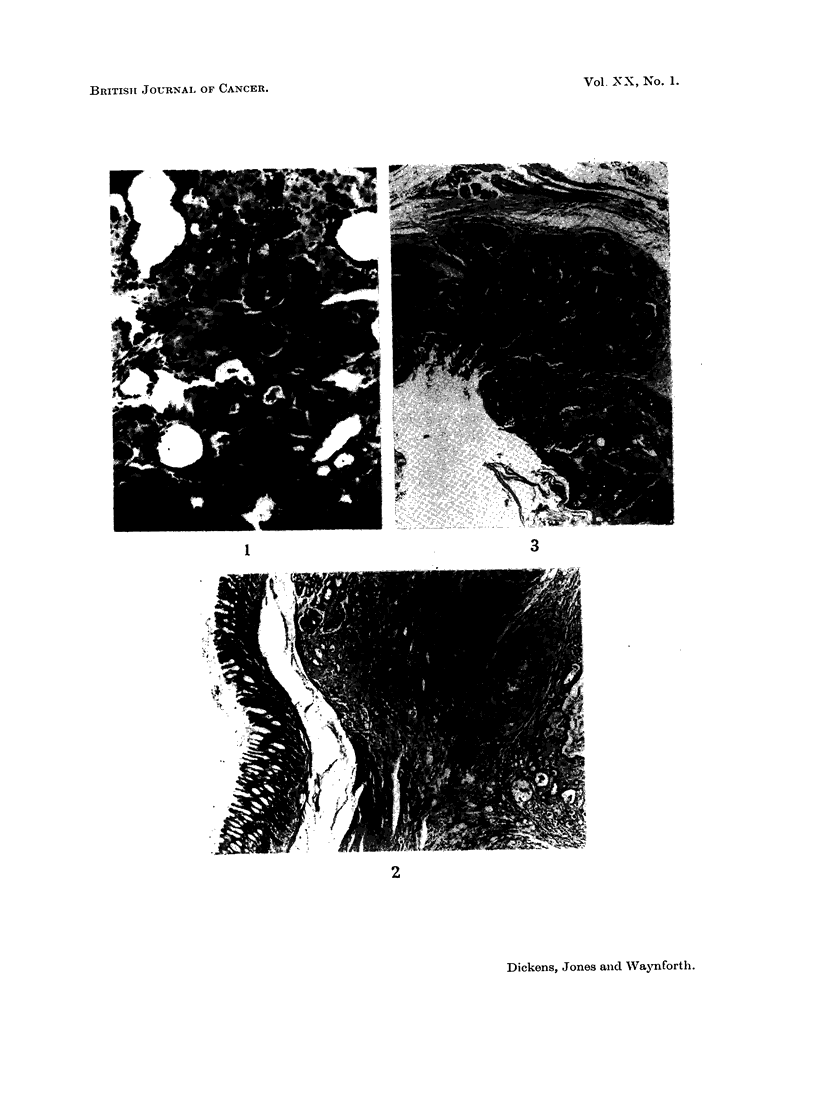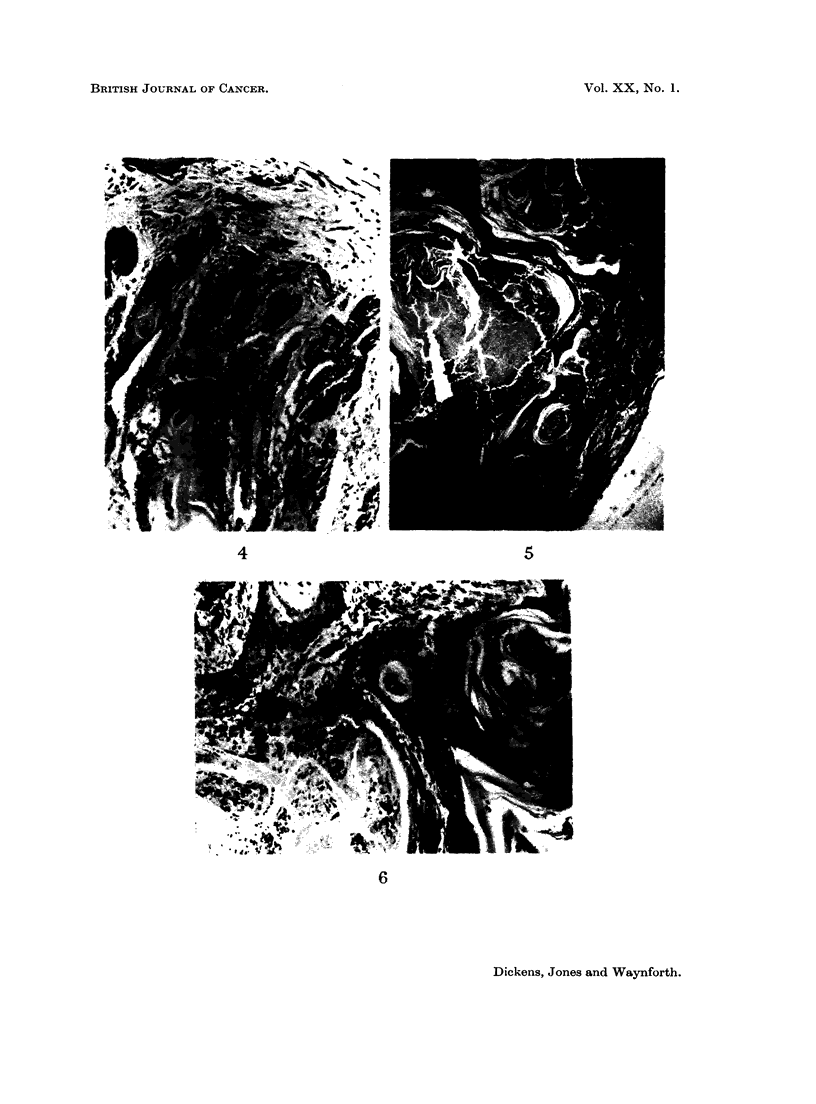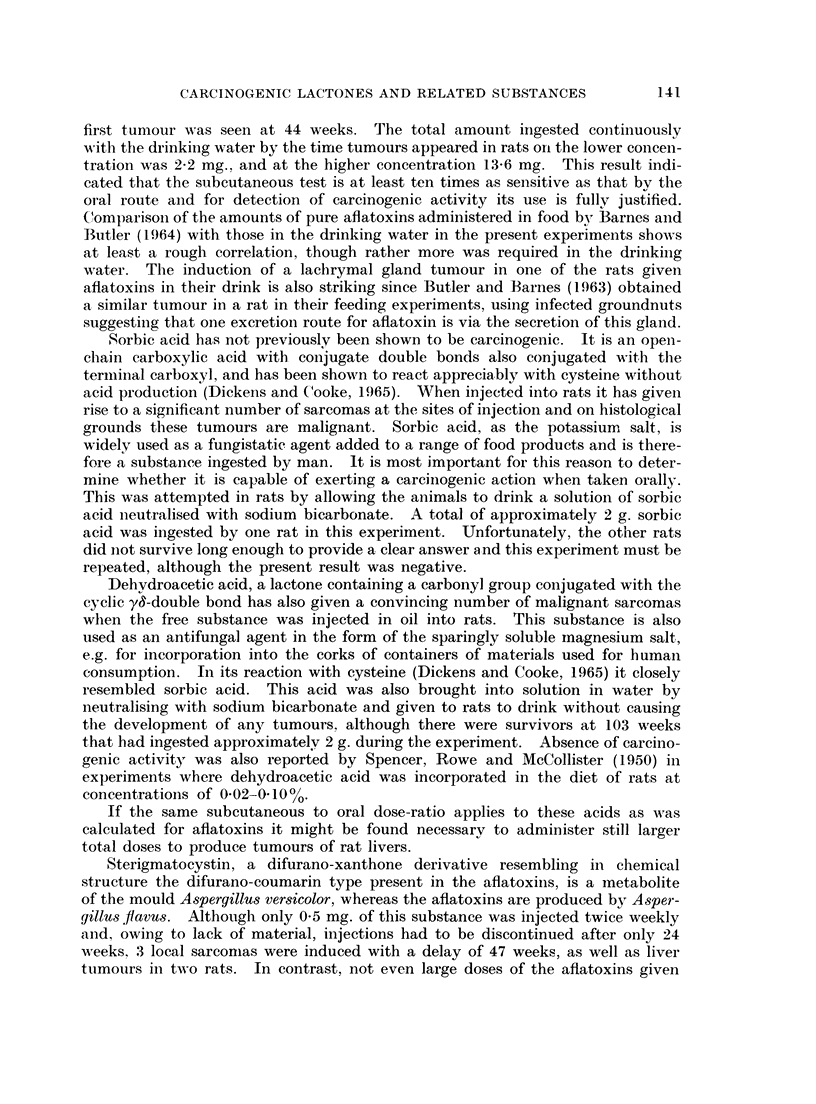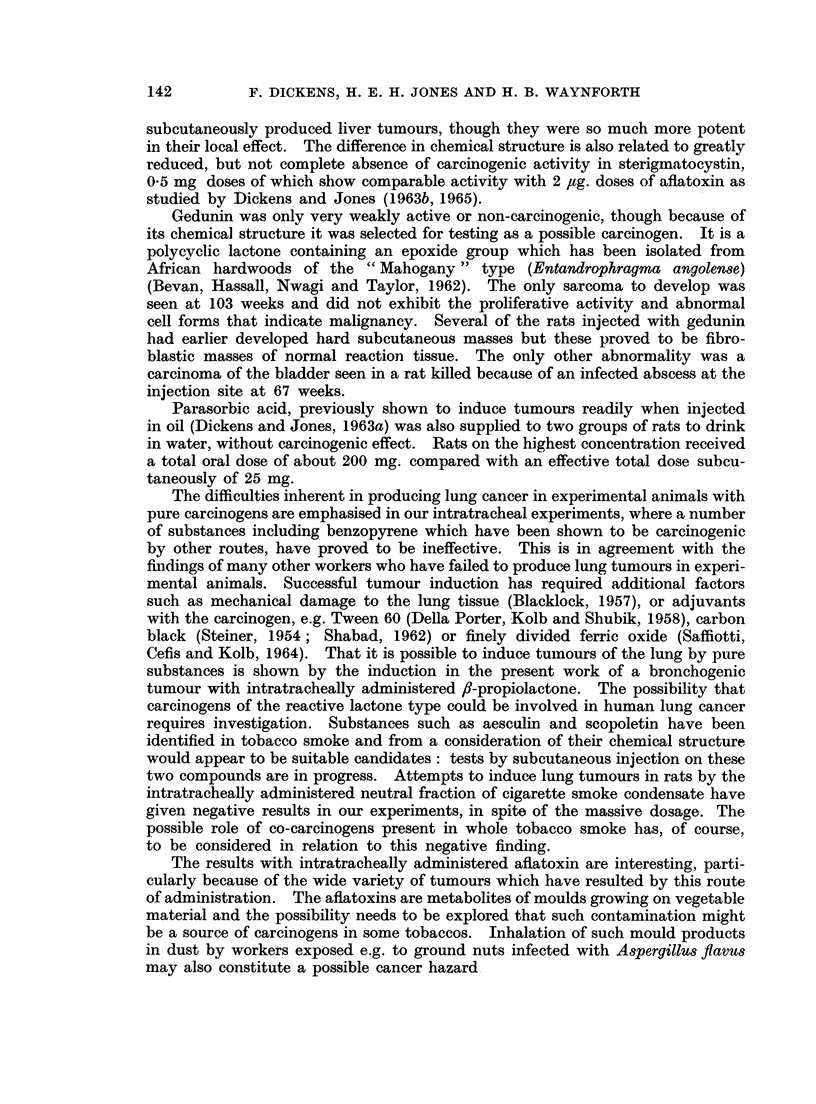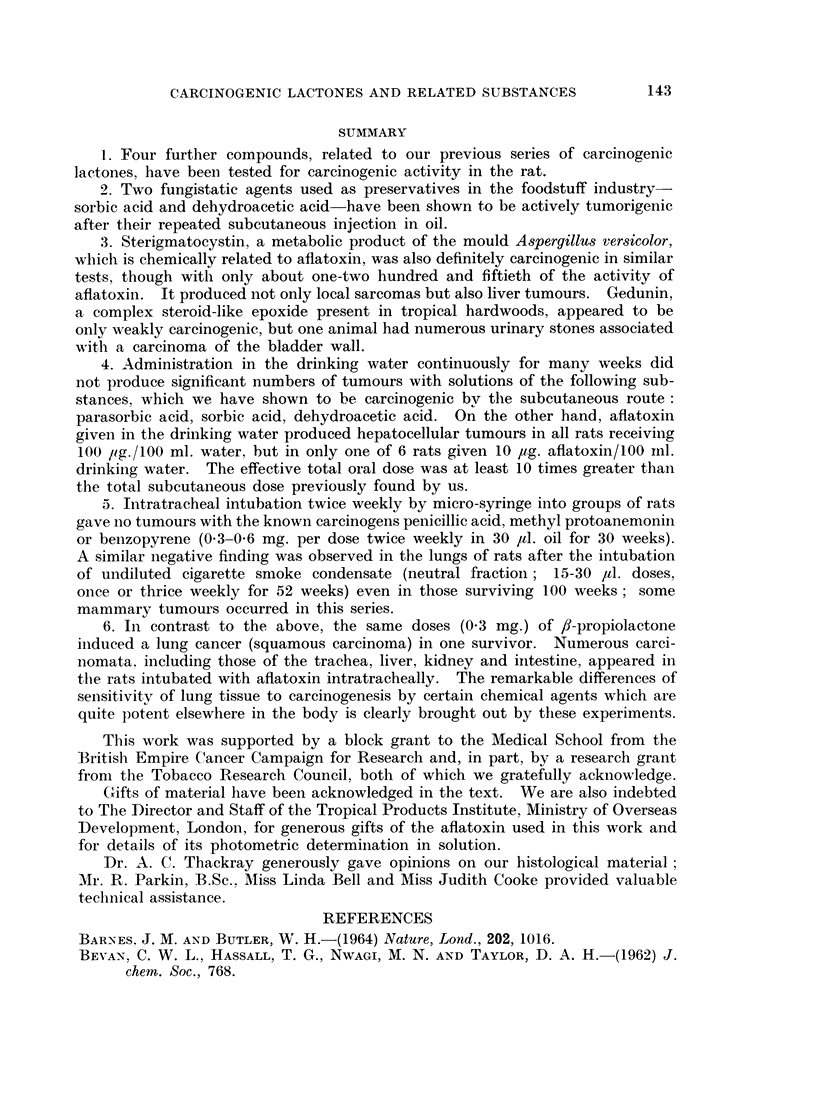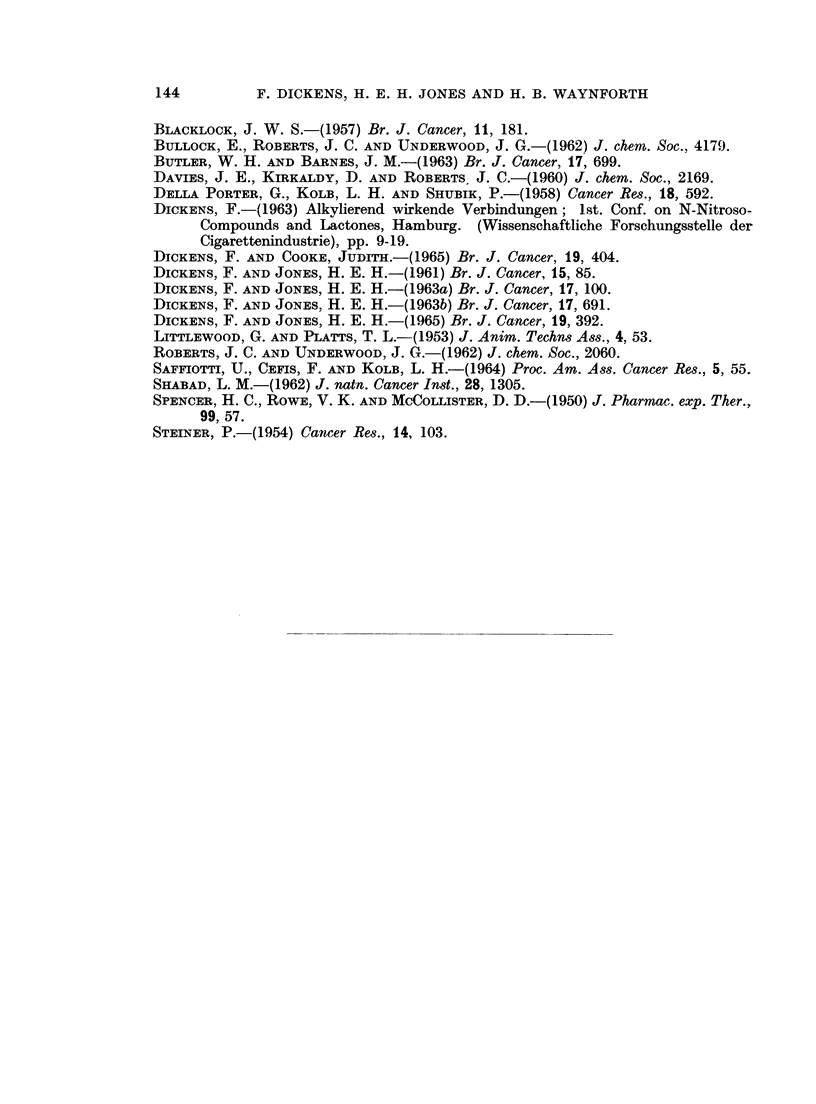# Oral, subcutaneous and intratracheal administration of carcinogenic lactones and related substances: the intratracheal administration of cigarette tar in the rat.

**DOI:** 10.1038/bjc.1966.15

**Published:** 1966-03

**Authors:** F. Dickens, H. E. Jones, H. B. Waynforth

## Abstract

**Images:**


### ORAL, SUBCUTANEOUS AND INTRATRACHEAL ADMINISTRATION OF CARCINOGENIC LACTONES AND RELATED SUBSTANCES: THE INTRATRACHEAL ADMINISTRATION OF CIGARETTE TAR IN THE RAT

F. DICKENS, H. E. H. JONES AND H. B. WAYNFORTH

*From the Courtauld Institute of Biochemistry, Middlesex Hospital Medical School, London W.1*

Received for publication January 6, 1966

It has now been possible to test 33 compounds with a lactonic or related structure, of which 25 have proved on subcutaneous injection to be capable of inducing the formation of tumours of the connective tissue elements of rats (Dickens and Jones, 1961; 1963*a*, *b*; 1965). The carcinogenic substances are for the most part characterised by chemical structures which indicate their reactive nature. This may be due to:

a. Strain in the lactone ring, particularly in the 4-membered rings.
b. Conjugation of double bonds, within and outside the ring, with the lactonic carbonyl group.
c. Conjugation of double bonds even in linear compounds.
d. Presence of the anhydride ring of dicarboxylic acids.

Where these features were absent, or where the substances caused death of the animals before tumours could be expected to appear, it was not possible to show a carcinogenic action in members of this series.

Dickens and Cooke (1965) studied the rates of hydrolysis and of interaction with cysteine of these substances and showed that, with the exception of aflatoxin and N-ethyl maleimide, the rates of reaction with cysteine could be correlated in a very approximate manner with their carcinogenic activity.

It is becoming clear that carcinogens belonging to this chemical class might be a pertinent factor in human cancer. Many compounds of this general type are widespread in animal and vegetable tissues, are produced by some saprophytic fungi infecting foodstuffs, and may possibly be produced during the burning of tobacco (Dickens, 1963). Some of them are useful antibiotics, and it is unfortunate that the very molecular structure which makes them active agents against bacteria and fungi is probably also that which makes them active as carcinogens. In relation to this problem it is important to know whether such compounds are carcinogenic not only when injected but when taken into the stomach and lungs, these being the routes by which some substances related to those dealt with in these studies are liable to be ingested by man.

This paper reports the results of testing four further substances chosen because of the similarity of their structure to other compounds which have already proved to be carcinogenic. They are sorbic acid and dehydroacetic acid—salts of which are widely used as fungistatic agents—sterigmatocystin, a mould metabolite, and gedunin, isolated from African hardwoods. Sorbic acid and dehydroacetic acid have also been administered by mouth, as well as some other substances which have previously been shown to be carcinogenic by injection. We also report the result of an experiment with a series of known carcinogens designed to investigate their effects on the lungs of rats. For comparison, cigarette smoke condensate has been similarly administered intratracheally to groups of rats over a long period.

### EXPERIMENTAL

### *Materials*

The sources and characteristics of aflatoxins $B_1$ and $G_1$, (+)-parasorbic acid, $\beta$-propiolactone, penicillic acid and methylprotoanemonin have already been described by Dickens and Jones (1961 ; 1963*a*, *b* ; 1965).

* *Sorbic acid* (trans-trans-2,4-hexadienoic acid, I), *dehydroacetic acid* (II) and *benzopyrene* were purchased from L. Light & Co. Colnbrook, Bucks.

*Sterigmatocystin* (III) was kindly provided by Professor J. C. Roberts, Department of Chemistry, University of Nottingham, through Dr. A. J. Feuell of the Tropical Products Institute, London (Davies, Kirkaldy and Roberts, 1960 ; Bullock, Roberts and Underwood, 1962 ; Roberts and Underwood, 1962).

*Gedunin* (IV) was a gift from Professor C. W. L. Bevan, Department of Chemistry, University of Ibadan, Nigeria (Bevan *et al.*, 1962).

*Cigarette smoke condensate* was made available by Dr. H. R. Bentley through the kind cooperation of the Tobacco Research Council, London (Director : Mr. G. F. Todd). The fraction used was the neutral material resulting from the standard T.R.C. fractionation process.

### *Animal experiments*

Twice-weekly subcutaneous injections of substances were made into the right flanks of groups of 6 rats weighing initially 100 g. for 65 weeks, or for as long as the supply lasted in the case of sterigmatocystin. The substances were injected as solutions in arachis oil, or as fine suspensions for sterigmatocystin and gedunin. A control group of 6 rats was injected with the oil alone. Sorbic acid, dehydroacetic acid and gedunin were injected in repeated doses of 2 mg. in 0·5 ml. oil and sterigmatocystin in doses of 0·5 mg. in 0·5 ml. oil. The mixed aflatoxins were injected at a dose of 2 $\mu$g. in 0·5 ml. oil and preliminary results relating to this group have already been reported by Dickens and Jones (1965).

Oral administration was achieved by supplying solutions of the substances to groups of 6 rats daily to drink for a period of 64 weeks. Concentrated stock solutions in water were stored at 4° and diluted with tap water to replenish the water bottles. Mixed aflatoxins were given to two groups of rats at concentrations of 100 $\mu$g. and 10·0 $\mu$g./100 ml. water, parasorbic acid to two groups at 1 mg. and 200 $\mu$g./100 ml. and sorbic acid and dehydroacetic acid to one group of rats each at 10 mg./100 ml. The latter two substances were neutralised with sodium bicarbonate to pH approximately 5 in order to bring them into solution. By laparotomy under ether anaesthesia the animals were examined at intervals for evidence of liver tumours. A complete search for tumours of all organs was made on animals when they died and on all those surviving at 100 weeks.

* For structural formulae I, II, III and IV see p. 140.

Solution of the sparingly soluble aflatoxins for use in the drinking water was achieved by adding the solution of 25 mg. mixed aflatoxins ($B_1 + G_1$) in 20 ml. warm absolute ethanol to a large volume of water (1980 ml.). After allowing to stand at 4° C. for several weeks, the amount remaining in solution was determined by measurement, against a blank containing the appropriate amount of ethanol, of the ultraviolet absorption. This corresponded to 80 per cent of the total amount originally taken, i.e. 10 $\mu$g./ml. instead of 12·5 $\mu$g./ml. This stock solution was then diluted daily with water 10 or 100 times for preparation of the solutions of aflatoxin as placed in the drinking bottles.

A technique for administering a substance by intubation directly into the trachea of a rat has been used to investigate the induction of lung cancer in treated animals. Modification of a technique (Littlewood and Platts, 1953) kindly described to us by the Staff of Messrs. Fison's Toxicological Laboratory, Saffron Walden, allowed administrations to be made at the rate of one rat per minute. The apparatus consisted of a sheet of perspex attached upright to a heavy wooden base and having its upper half inclined towards the operator. Rats, treated with atropine sulphate to prevent salivation were anaesthetised with ether and suspended by their fore limbs by loops of string. Suspension could be rapidly effected by fastening the loops round the limbs by a sliding piece of polythene tubing, provided that the two free ends of the loops passed through a hole in the perspex small enough to grip the double strand of string without tying. The head of the rat was pressed against the perspex by a rubber band which passed round it and under the upper incisors. For intubation, the animal's tongue was pulled forward and the inside of the mouth illuminated by a laryngoscope, of the type used for children, with the speculum removed. A curved, blunted serum needle, attached to a tuberculin syringe in an Agla micrometer, was passed between the vocal chords for approximately one inch into the trachea and the required amount of substance delivered. Up to 40 $\mu$l. of material was placed in this way at the lower end of the trachea and from this site it was expected to pass subsequently into the lungs. By fluorescent microscopy it has been possible to show that benzopyrene placed in the trachea passed into the lungs within a few hours while little or none appeared to be regurgitated into the mouth. Arachis oil alone was administered to 6 rats as a control by the same technique and both experimental and control rats were given tetracycline in the drinking water (100 mg./l.) throughout the course of the experiment to minimise the risk of intercurrent infections causing their death. Intratracheal administrations were made twice-weekly for 30 weeks with very good survival and the rats were examined when they died or at 100 weeks after the first administration, particularly for lesions of the respiratory and intestinal tracts. $\beta$-Propiolactone, penicillic acid, aflatoxins B and G, and benzopyrene were given intratracheally in doses of 0·3 mg., and methylprotoanemonin in doses of 0·6 mg. dissolved or suspended in 30 $\mu$l. of arachis oil, each to 6 male rats.

The undiluted neutral fraction of tobacco smoke condensate (30 $\mu$l. doses) was instilled intratracheally once weekly and 3 times weekly to separate groups, each of 10 female rats, throughout the course of one year. As controls of these groups, 6 female rats were treated with atropine, ether and tetracycline but were not given any intratracheal treatment.

All tissues that appeared to be abnormal by macroscopic observation were examined histologically as described by Dickens and Jones (1961).

### RESULTS

In the control injection experiment four rats survived for longer than 72 weeks, three eventually dying without a tumour, but one rat which died at 81 weeks carried a large sarcoma-like tumour, not histologically malignant, at the site of the injections (Table I). Two groups, each of 20 mice (not included in the Table) were also injected with the same arachis oil twice-weekly for 65 weeks but only 16 of these survived for longer than 69 weeks and in the survivors one malignant fibrosarcoma and one metastasising mammary adenoma were found at the site of injections. (Rats also withstood the repeated intratracheal administration of the oil extremely well without developing tumours in the lungs or elsewhere ; see Table III.)

Subcutaneous injections of sorbic acid, sterigmatocystin and dehydroacetic acid in oil have given rise to appreciable numbers of sarcomas in the injected rats (Table I), each group including tumours which, because of the marked proliferative activity and the presence of giant multinucleate cells, are considered to be malignant. Injections of sterigmatocystin also induced a hepatocellular tumour at 50 weeks and a cholangiocarcinoma at 62 weeks in rats which also bore local sarcomas. Gedunin injections gave rise to only one sarcomatous growth after a very long time and this on histological grounds was much less malignant. One rat at 67 weeks was killed because of an infected abscess at the site of the injections. The bladder of this animal contained 18 small calculi and the bladder wall was much thickened. When examined histologically it was seen that both surfaces of the bladder wall were covered with a squamous metaplasia by invasion from the inner surface. This is considered to be a carcinoma of the bladder for this reason.

TABLE I.—*The Carcinogenic Action of Compounds Administered Twice Weekly by Subcutaneous Injection in Oil to Groups of 6 Male Rats*

| Substance tested | Dose | Duration of treatment (weeks) | Earliest appearance of tumours (weeks) | Number of rats alive when first tumour seen | Number of rats with local tumours | Total period observed (weeks) | Other tumours found at autopsy |
|---|---|---|---|---|---|---|---|
| Arachis oil (controls) | (0·5 ml.) | 65 | 81 | 3 | 1 | 89 | None. |
| Aflatoxins ($B_1+G_1$) | 2 $\mu$g. | 65 | 44 | 6 | 5 | 73 | None. |
| Sorbic acid | 2 mg. | 65 | 82 | 6 | 5 | 97 | None. |
| Sterigmatocystin | 0·5 mg. | 24 | 47 | 6 | 3 | 65 | Hepatoma (50 weeks) Cholangioma (62 weeks). |
| Dehydroacetic acid | 2 mg. | 65 | 37 | 6 | 5 | 85 | None. |
| Gedunin | 2 mg. | 65 | 103 | 1 | 1 | 103 | None. |

Two microgram doses of the mixed aflatoxins injected in oil twice weekly for 65 weeks induced 5 malignant sarcomas in the 6 rats which were alive at the time of appearance of the first tumour. The 6th rat which was alive when a preliminary report (Dickens and Jones, 1965) was made of this result, eventually died without developing a tumour.

Of the substances administered to rats in their drinking water (Table II), only the mixed aflatoxins have shown themselves capable of inducing tumours,

TABLE II.—*The Effect of Various Substances with Known Carcinogenic Activity Added to the Drinking Water of Rats*

Groups of 6 male rats were used and administration continued for 64 weeks in each experiment

| Substance added to water | Concentration /100 ml. drinking water | Average weekly intake per rat (ml.) | Earliest appearance of liver tumours (weeks) | Number of tumours/ number of survivors | Other tumours found (time in weeks) and remarks |
|---|---|---|---|---|---|
| Aflatoxins ($B_1+G_1$) | 100 $\mu$g. | 307 | 39 | 6/6 | Lachrymal gland (53) |
| Aflatoxins ($B_1+G_1$) | 10 $\mu$g. | 356 | 66 | 1/5 | None |
| Parasorbic acid | 1 mg. | 309 | — | — | None |
| Parasorbic acid | 200 $\mu$g. | 220 | — | — | Leydig cell tumour (103) |
| Sorbic acid | 10 mg. | 312 | — | — | None—only one rat surviving at 64 weeks |
| Dehydroacetic acid | 10 mg. | 463 | — | — | None—liver necrosis in a rat alive at 103 weeks |

mainly of the liver but one also of the lachrymal gland. The liver tumours were first seen in rats given the larger dose when they were first examined by laparotomy at 39 weeks. Eventually all the rats drinking water containing 100 $\mu$g. of aflatoxins/100 ml. developed hepatocellular tumours. The lachrymal gland tumour weighing 1·5 g. was also found in one of the animals of this group at 53 weeks. Only one rat given drinking water containing the lower concentration of aflatoxins, 10·0 $\mu$g./100 ml., developed a liver tumour, found at laparotomy at 66 weeks. No liver tumours were seen in rats given parasorbic acid at 1 mg./100 ml. or at 200 $\mu$g./100 ml., or given sorbic acid or dehydroacetic acid at 10 mg./100 ml. to drink. One rat which had been given parasorbic acid (200 $\mu$g./100 ml.) to drink for 64 weeks was found to have a Leydig cell tumour when killed at 103 weeks and necrosis of the livers was seen in rats which survived to 103 weeks after drinking dehydroacetic acid (10 mg./100 ml.) for 64 weeks. Intercurrent disease affected some groups in this series adversely, so that of the rats given sorbic acid to drink five died quite early in the experiment with abscesses of the lungs, pericardium and peritoneum. Only one of these rats survived for the full treatment period and was killed at 64 weeks, but no tumours were found. Three rats given 200 $\mu$g. parasorbic acid/100 ml. water to drink also died of generalised infection at the same time, though those which survived eventually died at 87, 95 and 100 weeks. None of these developed tumours of the digestive tract or related organs, but there was a testicular tumour in the last survivor.

Following the intratracheal administration of penicillic acid (0·3 mg.), methyl protoanemonin (0·6 mg.) and benzopyrene (0·3 mg.) twice weekly for 30 weeks, and tobacco tar neutral fraction (30 $\mu$l.) once weekly or three times weekly for 52 weeks, no tumours were seen in the lungs even in rats which survived to 100 weeks (Table III). All the rats given the tobacco smoke condensate, and their controls, showed severe changes in the delicate alveolar tissue and some hyperplasia of the bronchiolar epithelium. There was also a great deal of infiltration by inflammatory cells and macrophages with abscesses developing particularly in the rats treated with the condensate. However, none of the rats showed metaplastic or malignant changes of the lung tissues. Mammary gland tumours were found at 74, 95 and 104 weeks in three rats treated with condensate and in

TABLE III.—*Intratracheal Administration to Rats*

| Substance administered | Dose (twice weekly unless stated) | Weeks administered | Appearance of lung tumours (weeks) | Time of deaths (weeks) | Other tumours (time in weeks) |
|---|---|---|---|---|---|
| Oil . . . | (30 µl.) | 30 | — | 62, 68, 72, 79, 80 | — |
| β-Propiolactone in oil (30 µl.) | 0·3 mg. | 30 | 72, Squamous carcinoma of bronchii | 12, 67, 72, 78, 89 and 100 | — |
| Penicillic acid in oil (30 µl.) | 0·3 mg. | 30 | — | 13, 16, 72, 85, 86, 92 | — |
| Aflatoxins in oil (30 µl.) | 0·3 mg. | 30 | 37, Tracheal squamous carcinoma. 52, ditto. 62, ditto. | | Hepatoma and cholangioma (49). Hepatoma, renal adenoma, carcinoma of pylorus (49). Hepatoma (52). Hepatoma (62). |
| Methylproto-anemonin in oil (30 µl.) | 0·6 mg. | 30 | — | 10, 63, 100, 100, 100, 100 | — |
| Benzopyrene in oil (30 µl.) | 0·3 mg. | — | — | 8, 25, 47, 100, 100 | — |
| Controls without oil | — | — | — | 16, 16, 28, 88, 99, 102 | Thymoma (88) Mammary tumour (99) |
| Neutral fraction of cigarette smoke condensate | 15–30 µl. once weekly | 52 | — | 23, 43, 63, 63, 74, 74, 84, 101, 104, 104 | Mammary tumour (74) |
| Neutral fraction of cigarette smoke condensate | 15–30 µl. 3 times weekly | 52 | — | 23, 39, 51, 64, 88, 95, 101, 104, 104, 104 | Mammary tumours (95 and 104) Uterine tumour (104) |

one of their controls at 99 weeks. There was also a tumour of the uterus at 104 weeks present in one rat treated with the tobacco tar, and a thymoma in one of the control rats which died at 88 weeks.

The aflatoxins, administered intratracheally, were effective carcinogens and produced a variety of tumours. In the trachea of 3 rats, invading squamous carcinomas (Fig. 3 and 4) developed eventually killing the animals by occluding the air passage at 37, 52 and 62 weeks respectively. The latter two animals also suffered from liver cancers (Fig. 1), as also did two other rats which died at 49 weeks. One of these also had a renal adenoma and a globular growth on the pyloric portion of the intestine which has been identified as a carcinoma (Fig. 2). β-Propiolactone by the same route induced a lung cancer in one of the rats. This tumour (Fig. 5 and 6) involved only one lobe of the lung and was an advanced, keratinising squamous cell carcinoma which killed the animal at 72 weeks. Three other rats treated intratracheally with β-propiolactone survived to 78, 89 and 100 weeks but without developing any tumours.

### DISCUSSION

Previously (Dickens and Jones, 1965) 24 rats had been given repeated subcutaneous injections of arachis oil without developing tumours at the injection site although they had been observed for periods of up to 108 weeks. In the current control experiment (where oil from the same importer, but a different batch, was used) the appearance of a tumour resembling a fibrosarcoma, though not with histological characteristics of malignancy, following repeated injections of the oil gives an overall incidence of one tumour in a total of 28 rats. This is a somewhat lower incidence than in similar control experiments with the same oil that we have carried out with a random bred strain of albino mice and indicates the suitability of the rat as a test animal.

I

II

III

IV

Aflatoxin by the three routes of administration employed has shown itself to be a most effective carcinogen, though the dose requirement by different routes was not the same. Following subcutaneous injection twice weekly in 2 μg. doses a total of 166 μg. of the aflatoxins had been administered by the time the

---

EXPLANATION OF PLATES

FIG. 1.— Mixed liver tumour in a rat given 300 μg. aflatoxins ($B_1$ + $G_1$) intratracheally twice-weekly for 30 weeks. The liver tumour was found at death 19 weeks after the last administration of aflatoxins. × 100.

FIG. 2.—Adenocarcinoma of the intestine found in a rat which died 19 weeks after 30 weeks of treatment with 300 μg. aflatoxins ($B_1$ + $G_1$) intratracheally twice-weekly. Normal mucosa to the left of the picture, invading adenocarinoma to the right. × 23.

FIG. 3.—Squamous carcinoma of the tracheal epithelium of a rat invading the tracheal wall between the cartilaginous rings. This tumour was seen in a rat treated with twice weekly intratracheal doses of aflatoxins ($B_1$ + $G_1$, 300 μg.) for 30 weeks when it died 32 weeks later. × 23.

FIG. 4.—Squamous metaplasia and carcinoma in a tumour or the tracheal epithelium of a rat treated intratracheally with aflatoxins ($B_1$ + $G_1$, 300 μg.) × 100.

FIG. 5.—Keratinising squamous carcinoma of rat lung found at death in an animal 42 weeks after cessation of 30 weeks of intratracheal administration of 300 μg. β-propiolactone twice weekly. × 23.

FIG. 6.—Higher magnification of one area of the lung carcinoma shown in Fig. 5. × 100.

1

3

2

4

5

6

first tumour was seen at 44 weeks. The total amount ingested continuously with the drinking water by the time tumours appeared in rats on the lower concentration was 2·2 mg., and at the higher concentration 13·6 mg. This result indicated that the subcutaneous test is at least ten times as sensitive as that by the oral route and for detection of carcinogenic activity its use is fully justified. Comparison of the amounts of pure aflatoxins administered in food by Barnes and Butler (1964) with those in the drinking water in the present experiments shows at least a rough correlation, though rather more was required in the drinking water. The induction of a lachrymal gland tumour in one of the rats given aflatoxins in their drink is also striking since Butler and Barnes (1963) obtained a similar tumour in a rat in their feeding experiments, using infected groundnuts suggesting that one excretion route for aflatoxin is via the secretion of this gland.

Sorbic acid has not previously been shown to be carcinogenic. It is an open-chain carboxylic acid with conjugate double bonds also conjugated with the terminal carboxyl, and has been shown to react appreciably with cysteine without acid production (Dickens and Cooke, 1965). When injected into rats it has given rise to a significant number of sarcomas at the sites of injection and on histological grounds these tumours are malignant. Sorbic acid, as the potassium salt, is widely used as a fungistatic agent added to a range of food products and is therefore a substance ingested by man. It is most important for this reason to determine whether it is capable of exerting a carcinogenic action when taken orally. This was attempted in rats by allowing the animals to drink a solution of sorbic acid neutralised with sodium bicarbonate. A total of approximately 2 g. sorbic acid was ingested by one rat in this experiment. Unfortunately, the other rats did not survive long enough to provide a clear answer and this experiment must be repeated, although the present result was negative.

Dehydroacetic acid, a lactone containing a carbonyl group conjugated with the cyclic $\gamma\delta$-double bond has also given a convincing number of malignant sarcomas when the free substance was injected in oil into rats. This substance is also used as an antifungal agent in the form of the sparingly soluble magnesium salt, e.g. for incorporation into the corks of containers of materials used for human consumption. In its reaction with cysteine (Dickens and Cooke, 1965) it closely resembled sorbic acid. This acid was also brought into solution in water by neutralising with sodium bicarbonate and given to rats to drink without causing the development of any tumours, although there were survivors at 103 weeks that had ingested approximately 2 g. during the experiment. Absence of carcinogenic activity was also reported by Spencer, Rowe and McCollister (1950) in experiments where dehydroacetic acid was incorporated in the diet of rats at concentrations of 0·02–0·10%.

If the same subcutaneous to oral dose-ratio applies to these acids as was calculated for aflatoxins it might be found necessary to administer still larger total doses to produce tumours of rat livers.

Sterigmatocystin, a difurano-xanthone derivative resembling in chemical structure the difurano-coumarin type present in the aflatoxins, is a metabolite of the mould *Aspergillus versicolor*, whereas the aflatoxins are produced by *Aspergillus flavus*. Although only 0·5 mg. of this substance was injected twice weekly and, owing to lack of material, injections had to be discontinued after only 24 weeks, 3 local sarcomas were induced with a delay of 47 weeks, as well as liver tumours in two rats. In contrast, not even large doses of the aflatoxins given subcutaneously produced liver tumours, though they were so much more potent in their local effect. The difference in chemical structure is also related to greatly reduced, but not complete absence of carcinogenic activity in sterigmatocystin, 0·5 mg doses of which show comparable activity with 2 $\mu$g. doses of aflatoxin as studied by Dickens and Jones (1963*b*, 1965).

Gedunin was only very weakly active or non-carcinogenic, though because of its chemical structure it was selected for testing as a possible carcinogen. It is a polycyclic lactone containing an epoxide group which has been isolated from African hardwoods of the "Mahogany" type (*Entandrophragma angolense*) (Bevan, Hassall, Nwagi and Taylor, 1962). The only sarcoma to develop was seen at 103 weeks and did not exhibit the proliferative activity and abnormal cell forms that indicate malignancy. Several of the rats injected with gedunin had earlier developed hard subcutaneous masses but these proved to be fibroblastic masses of normal reaction tissue. The only other abnormality was a carcinoma of the bladder seen in a rat killed because of an infected abscess at the injection site at 67 weeks.

Parasorbic acid, previously shown to induce tumours readily when injected in oil (Dickens and Jones, 1963*a*) was also supplied to two groups of rats to drink in water, without carcinogenic effect. Rats on the highest concentration received a total oral dose of about 200 mg. compared with an effective total dose subcutaneously of 25 mg.

The difficulties inherent in producing lung cancer in experimental animals with pure carcinogens are emphasised in our intratracheal experiments, where a number of substances including benzopyrene which have been shown to be carcinogenic by other routes, have proved to be ineffective. This is in agreement with the findings of many other workers who have failed to produce lung tumours in experimental animals. Successful tumour induction has required additional factors such as mechanical damage to the lung tissue (Blacklock, 1957), or adjuvants with the carcinogen, e.g. Tween 60 (Della Porter, Kolb and Shubik, 1958), carbon black (Steiner, 1954; Shabad, 1962) or finely divided ferric oxide (Saffiotti, Cefis and Kolb, 1964). That it is possible to induce tumours of the lung by pure substances is shown by the induction in the present work of a bronchogenic tumour with intratracheally administered $\beta$-propiolactone. The possibility that carcinogens of the reactive lactone type could be involved in human lung cancer requires investigation. Substances such as aesculin and scopoletin have been identified in tobacco smoke and from a consideration of their chemical structure would appear to be suitable candidates: tests by subcutaneous injection on these two compounds are in progress. Attempts to induce lung tumours in rats by the intratracheally administered neutral fraction of cigarette smoke condensate have given negative results in our experiments, in spite of the massive dosage. The possible role of co-carcinogens present in whole tobacco smoke has, of course, to be considered in relation to this negative finding.

The results with intratracheally administered aflatoxin are interesting, particularly because of the wide variety of tumours which have resulted by this route of administration. The aflatoxins are metabolites of moulds growing on vegetable material and the possibility needs to be explored that such contamination might be a source of carcinogens in some tobaccos. Inhalation of such mould products in dust by workers exposed e.g. to ground nuts infected with *Aspergillus flavus* may also constitute a possible cancer hazard

### SUMMARY

1. Four further compounds, related to our previous series of carcinogenic lactones, have been tested for carcinogenic activity in the rat.

2. Two fungistatic agents used as preservatives in the foodstuff industry—sorbic acid and dehydroacetic acid—have been shown to be actively tumorigenic after their repeated subcutaneous injection in oil.

3. Sterigmatocystin, a metabolic product of the mould *Aspergillus versicolor*, which is chemically related to aflatoxin, was also definitely carcinogenic in similar tests, though with only about one-two hundred and fiftieth of the activity of aflatoxin. It produced not only local sarcomas but also liver tumours. Gedunin, a complex steroid-like epoxide present in tropical hardwoods, appeared to be only weakly carcinogenic, but one animal had numerous urinary stones associated with a carcinoma of the bladder wall.

4. Administration in the drinking water continuously for many weeks did not produce significant numbers of tumours with solutions of the following substances, which we have shown to be carcinogenic by the subcutaneous route : parasorbic acid, sorbic acid, dehydroacetic acid. On the other hand, aflatoxin given in the drinking water produced hepatocellular tumours in all rats receiving 100 $\mu$g./100 ml. water, but in only one of 6 rats given 10 $\mu$g. aflatoxin/100 ml. drinking water. The effective total oral dose was at least 10 times greater than the total subcutaneous dose previously found by us.

5. Intratracheal intubation twice weekly by micro-syringe into groups of rats gave no tumours with the known carcinogens penicillic acid, methyl protoanemonin or benzopyrene (0·3–0·6 mg. per dose twice weekly in 30 $\mu$l. oil for 30 weeks). A similar negative finding was observed in the lungs of rats after the intubation of undiluted cigarette smoke condensate (neutral fraction ; 15-30 $\mu$l. doses, once or thrice weekly for 52 weeks) even in those surviving 100 weeks ; some mammary tumours occurred in this series.

6. In contrast to the above, the same doses (0·3 mg.) of $\beta$-propiolactone induced a lung cancer (squamous carcinoma) in one survivor. Numerous carcinomata, including those of the trachea, liver, kidney and intestine, appeared in the rats intubated with aflatoxin intratracheally. The remarkable differences of sensitivity of lung tissue to carcinogenesis by certain chemical agents which are quite potent elsewhere in the body is clearly brought out by these experiments.

This work was supported by a block grant to the Medical School from the British Empire Cancer Campaign for Research and, in part, by a research grant from the Tobacco Research Council, both of which we gratefully acknowledge.

Gifts of material have been acknowledged in the text. We are also indebted to The Director and Staff of the Tropical Products Institute, Ministry of Overseas Development, London, for generous gifts of the aflatoxin used in this work and for details of its photometric determination in solution.

Dr. A. C. Thackray generously gave opinions on our histological material ; Mr. R. Parkin, B.Sc., Miss Linda Bell and Miss Judith Cooke provided valuable technical assistance.

### REFERENCES

Barnes, J. M. and Butler, W. H.—(1964) *Nature, Lond.*, **202**, 1016.

Bevan, C. W. L., Hassall, T. G., Nwagi, M. N. and Taylor, D. A. H.—(1962) *J. chem. Soc.*, 768.

BLACKLOCK, J. W. S.—(1957) *Br. J. Cancer*, **11**, 181.
BULLOCK, E., ROBERTS, J. C. AND UNDERWOOD, J. G.—(1962) *J. chem. Soc.*, 4179.
BUTLER, W. H. AND BARNES, J. M.—(1963) *Br. J. Cancer*, **17**, 699.
DAVIES, J. E., KIRKALDY, D. AND ROBERTS, J. C.—(1960) *J. chem. Soc.*, 2169.
DELLA PORTER, G., KOLB, L. H. AND SHUBIK, P.—(1958) *Cancer Res.*, **18**, 592.
DICKENS, F.—(1963) Alkylierend wirkende Verbindungen; 1st. Conf. on N-Nitroso-Compounds and Lactones, Hamburg. (Wissenschaftliche Forschungsstelle der Cigarettenindustrie), pp. 9-19.
DICKENS, F. AND COOKE, JUDITH.—(1965) *Br. J. Cancer*, **19**, 404.
DICKENS, F. AND JONES, H. E. H.—(1961) *Br. J. Cancer*, **15**, 85.
DICKENS, F. AND JONES, H. E. H.—(1963*a*) *Br. J. Cancer*, **17**, 100.
DICKENS, F. AND JONES, H. E. H.—(1963*b*) *Br. J. Cancer*, **17**, 691.
DICKENS, F. AND JONES, H. E. H.—(1965) *Br. J. Cancer*, **19**, 392.
LITTLEWOOD, G. AND PLATTS, T. L.—(1953) *J. Anim. Techns Ass.*, **4**, 53.
ROBERTS, J. C. AND UNDERWOOD, J. G.—(1962) *J. chem. Soc.*, 2060.
SAFFIOTTI, U., CEFIS, F. AND KOLB, L. H.—(1964) *Proc. Am. Ass. Cancer Res.*, **5**, 55.
SHABAD, L. M.—(1962) *J. natn. Cancer Inst.*, **28**, 1305.
SPENCER, H. C., ROWE, V. K. AND MCCOLLISTER, D. D.—(1950) *J. Pharmac. exp. Ther.*, **99**, 57.
STEINER, P.—(1954) *Cancer Res.*, **14**, 103.